# What Are the Effects of Electronic Cigarettes on Lung Function Compared to Non-Electronic Cigarettes? A Systematic Analysis

**DOI:** 10.3389/ijph.2022.1604989

**Published:** 2022-09-30

**Authors:** Yumeng Song, Xin Li, Chaoxiu Li, Shuang Xu, Yong Liu, Xiaomei Wu

**Affiliations:** ^1^ Department of Clinical Epidemiology and Center of Evidence Based Medicine, The First Hospital of China Medical University, Shenyang, China; ^2^ Department of Infectious Diseases, The First Hospital of China Medical University, Shenyang, China; ^3^ Department of library of China Medical University, Library of China Medical University, Shenyang, China; ^4^ Department of Stomatology, Dalian University, Dalian, China; ^5^ Periodontology and Preventive Dentistry, Saarland University, Saarbrücken, Germany

**Keywords:** electronic cigarettes and pulmonary function electronic nicotine delivery systems, vaping, pulmonary function, pulmonary diffusion function, pulmonary ventilation function

## Abstract

**Objective:** The effects of e-cigarettes on lung function were compared between the e-cigarette and the non-e-cigarette group, as well as self-changes after inhaling e-cigarettes.

**Method:** From March 1st, 2022, relevant literature was selected from four databases through a predefined retrieval strategy. Strict literature screening and quality evaluation were conducted. The study followed PRISMA guidelines.

**Results:** Our results showed that CO (SMD: −1.48, 95%: −2.82–0.15) and FeNO (SMD: −0.66, 95%: −1.32, −0.01) were significantly decreased after e-cigarette usage. Only asthmatic smokers showed a statistically significant increase in flow resistance after inhaling e-cigarettes. Conversely, the decrease of FEV1/FVC% in the non-e-cigarette groups exceeded that in the e-cigarette group (SMD:1.18, 95%: 0.11–2.26). The degree of O_2_ saturation decrease was also less than that for the cigarette groups (SMD:0.32, 95%: 0.04–0.59), especially when compared to the conventional cigarette group (SMD:0.56, 95%: 0.04–1.08).

**Conclusion:** The current findings indicate that short-term e-cigarette inhalation has a similar (but not significant) effect on lung function, as compared with non-e-cigarettes. More clinical studies are needed to explore the safety of inhaling e-cigarettes, especially in vulnerable populations.

## Introduction

Electronic cigarettes (e-cigarettes/ECs, their usage also known as “vaping”) are devices that produce aerosols by heating liquids containing nicotine and other additives [1]. All e-cigarette systems are comprised of a battery, a cartridge/tank with liquid (e-liquid), and finally, an atomizer containing a wick, coil, and heating element. The wick draws the e-liquid into the coil, and when the device is activated, the e-liquid is heated, and the aerosol is then inhaled by the e-cigarette user [2]. Compared with the traditional cigarette, e-cigarette is billed as more healthy, more easily accepted by the society. E-cigarettes are marketed as a smoking cessation aid, although the effectiveness of e-cigarettes is not well understood [3]. Currently, e-cigarettes are considered a “new form of smoking”, and they are increasingly favoured by the young.

Indeed, the use of e-cigarettes is spreading rapidly across the world, especially in North America and the UK [4]. In the United States, 27.5% of high-school students are currently e-cigarette users, and about 13% of adults have used e-cigarettes [5]. This prevalence has raised concerns among a broad range of public-health researchers and research scientists [5], who consider the phenomenon a potentially important public-health problem. Food and Drug Administration (FDA) announced that it would begin to regulate E-cigarettes as tobacco products. Under the proposed rules, the FDA would ban the sale of the products to persons under the age of 18 [3]. Recent studies have highlighted the effects of e-cigarettes on the cardiovascular system [6, 7], oral health [8, 9], the immune system [10], and other systems [11].

Regarding the effects of e-cigarettes on the respiratory system, only one recent meta-analysis has so far reported on the physiological effects of acute electronic-cigarette use in humans. Moreover, even that study addressed only four lung-function indicators (FEV1, FVC, FEV1/FVC, FeNO) [7]. Pulmonary function testing is fundamental to clinical decision making, not only for patients with lung disease but also for a wide range of subjects who have symptoms of dyspnea, require chest or abdominal surgery, or may require screening. Specific tests include lung volume and airflow rate, diffusion capacity and airway resistance [12]. We will thus conduct a systematic analysis of all relevant studies published, so far, that encompass indicators for that area. This review mainly focuses on the following two aspects: 1) what is the difference between the e-cigarette uses and the cigarette smokers as well as between the non-users, on the affect of lung function; 2) What is the change of lung function in different populations after exposure to e-cigarette?

## Methods

This systematic review was conducted according to the guidelines of Preferred Reporting Items for Systematic Reviews and Meta-Analyses (PRISMA) [13] (Supplementary Table S1).

### Search Strategy

Four databases (PubMed, Web of Science, Embase and Cochrane) were searched, with a chronological end-point of 1st January 2022. The medical terms (Mesh) used were: “electronic cigarette,” “e-cig” and “e-cigarette,” in conjunction with “pulmonary,” “lung” and “respiratory.” Studies were also identified by searching the references of previously included articles (Supplementary Table S2).

### Study Selection

The titles and abstracts of the initially retrieved literature were screened, and then all potentially relevant articles were evaluated based on the full text. The criteria deployed were (1) the article reported on the relationship between e-cigarettes and indicators of pulmonary function or flow resistance, or data was provided to calculate the corresponding estimates; (2) the article comprised original human research; (3) if more than one article originated with the same research team, the latest or highest-quality text was adopted. If an article did not meet the above criteria, it was not considered. All differences regarding the study selection were adjudicated by the authors.

### Data Extraction

Two reviewers independently extracted data, double-checked the available data, and completed a standardized table *via* Microsoft Excel 2016. The following data were extracted: first author, year of publication, area, design, source of population, and baseline characteristics of sample population (age, gender, etc). Means and standard deviations (SDs) for the outcomes included in the systematic analysis (i.e., pulmonary ventilation capacity flow-resistance indicators) were recorded from cumulative, published data. Pulmonary ventilation capacity included FEV1 (forced expiratory volume in 1s), FEV1%, FVC (forced vital capacity), FVC%, FEV1/FVC% (forced expiratory volume in 1s to forced vital capacity), TV (tidal volume), TLC (total lung capacity), MEF25 (maximal expiratory flow at 25% of FVC), PEF (peak expiratory flow), and PEF%. Exhaled CO level (exhaled carbon-monoxide level), FeNO (fractional exhaled nitric oxide), and O_2_ saturation. Respiratory flow resistance includes respiratory impedance at 5 Hz (Z5Hz), flow resistance at 5, 10, 19 and 20 Hz (R5Hz, R10Hz, R19 Hz and R20Hz, respectively), the difference of R5 Hz and R19 Hz (R5-19 Hz), and the difference of R5 Hz and R20 Hz (R5-20 Hz). Respiratory-system reactance at 5 Hz and 20 Hz (X5, X10and X20), resonant frequency (fres) and reactance area (AX) were evaluated using an impulse oscillometry system (IOS).

### Literature Quality Assessment

Two reviewers independently assessed the risk of bias in the included studies. We used the Cochrane tool to assess the risk of bias of randomized, controlled trials (RCTs) [14]. For non-randomized intervention studies, we used the ROBINS-I tool (Cochrane Risk of Bias Assessment Tool for Non-Randomized Studies of Interventions) [15].

### Statistical Analysis

For each eligible item of literature included in the systematic analysis, we used the mean difference (MD) with a 95% confidence interval (CI), to investigate the pooled MD. The difference in MD for lung-function indicators was included in the quantitative synthesis, including 1) we compared changes in e-cigarette users with those in cigarette smoker groups (cigarette and dual-use groups) and then e-cigarette users with non-users (The non-users comprised the cessation group and e-cigarette use without nicotine, without e-liquid and without an e-cigarette cartridge, i.e. inactive devices.). 2) On this basis, we analyzed changes in lung function indices after e-cigarette exposure in different populations of interest (healthy smokers, nonsmokers, and asthmatic smokers). Smokers are defined as having smoked ≥1 cigarette in the past 30 days [16]. “Healthy” means those without any including the occurrence of any chronic diseases, history of lung conditions (e.g.,: asthma or bronchial hyperactivity in childhood), presence of any allergic diseases, medication intake within the last 2 weeks, acute illnesses or infections in the last 2 weeks, influenza vaccination in the last 2 weeks, or current pregnancy or lactation [17]. Non-smokers had no history of exposure to tobacco products or e-cigarette vapors [18]. Asthma patients were all diagnosed with asthma and had airway hyperresponsiveness, as shown by a positive methacholine challenge. They are classified and medicated according to the Global Initiative on Asthma (GINA) [16, 19].

We used the Chi-Square-Based-Q test to evaluate heterogeneity among the individual studies. The presence of heterogeneity was evaluated *via* the I^2^ and Q statistic. A random-effects model was used to obtain the pooled MD and 95% CI, because of clinical and methodological heterogeneity, such as differences in study design, the baseline characteristics of patients, and/or the implementation process of intervention measures. A sensitivity analysis was used to assess the stability of the results. Begg’s test and Egger’s test were used to evaluate publication bias. If publication bias was found to exist, the result was adjusted *via* the trim-and-fill method.

## Result

### Literature Search and Quality Assessment

**FIGURE 1:**
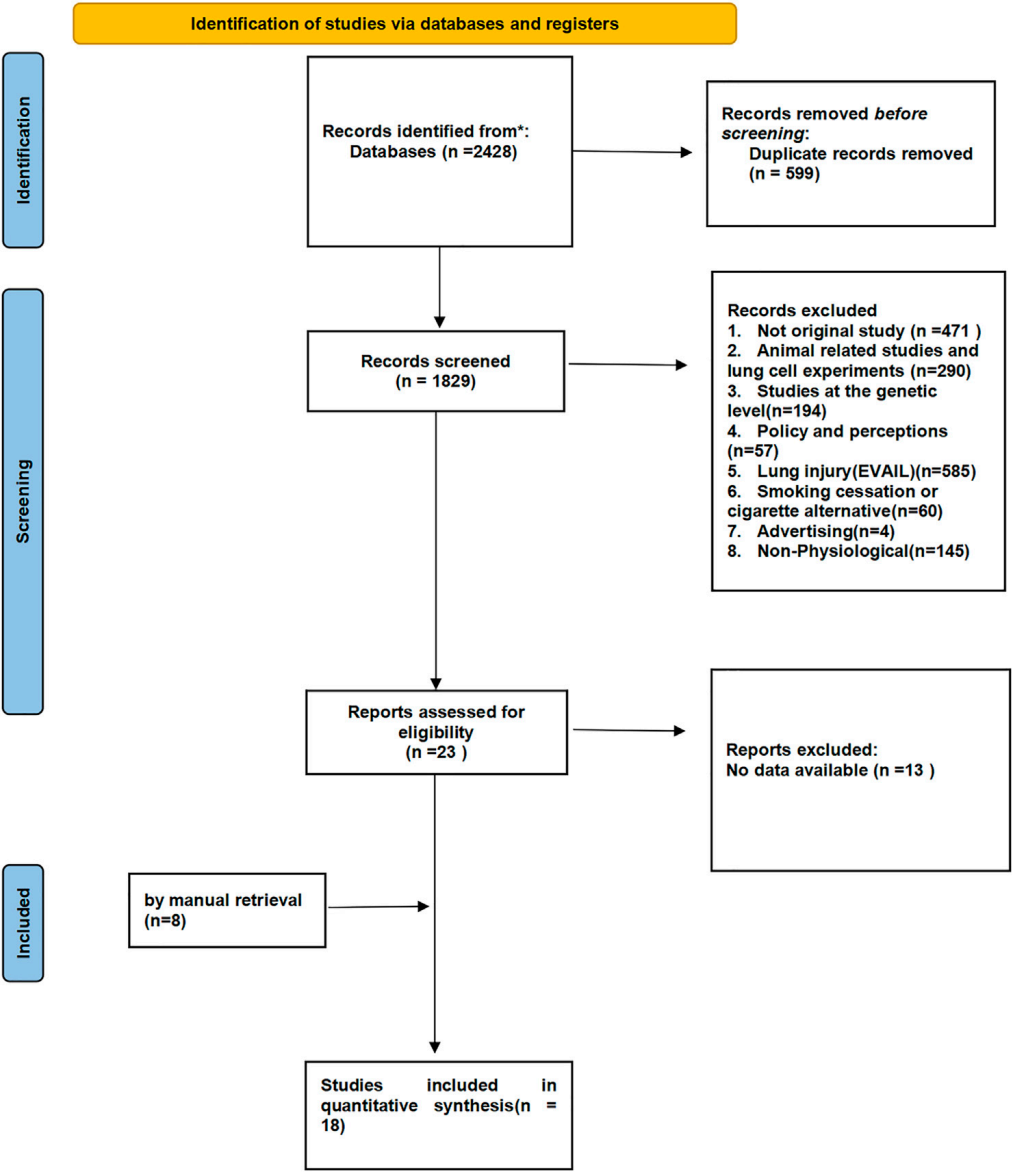
PRISMA flow chart of literature retrieval and selection (North America and Europe, 2012–2020).

We retrieved a total of 2,428 articles, and 599 duplicates were excluded after screening. Thus, 1829 qualified articles were extracted for full-text review. The majority of the excluded articles addressed e-cigarette prevalence, policies, perceptions, the efficiency of smoking cessation, and/or lung injury, or they did not mention physiological parameters (see Figure 1 for details). Finally, a total of 18 articles were eligible for inclusion in the systematic analysis.

The results of the literature quality assessment of randomized and non-randomized trials are shown in Supplementary Table S3 and Supplementary Figure S1.

### Study Characteristics

Details of the 18 individual studies included in the systematic analysis are summarized in Table 1. The number of participants ranged from 10 to 408. Sixteen of the studies explored the effects of e-cigarette inhalation on lung function within 1 month (exposure 5 min to 1 month), while two studies examined the effects on lung function after one and 3 months of e-cigarette use (exposure 1–24 months). The nicotine concentration in the e-cigarettes used ranged from 0.8–24 mg/ml. The mean age of the subjects ranged from 22.6–58 years, and males accounted for 32%–100% (Table 1).

**TABLE 1:** Summary of included studies (North America and Europe, 2012–2020).

Author	Year	Area	Source of population	Population	Number of subjects	Control	Nicotine (mg/ml)	Intervention time	Measurement time	Gender (male %)	Age (mean)	Smoking years	Smoking quantity
Kotoulas, S [14]	2020	Greece	Population-base	Healthy smokers and asthmatic smokers	25	N/A	N/A	5 min	Immediately	32	39.88	N/A	pack/years: 15.04 ± 16.22
Brożek, G. M [15]	2019	Poland	Population-base	Healthy smokers and non-smoker	120	Dual use/CC/EC without e-liquid	0.6	5 min	Immediately/30 min	59.2	22.6	Use CC: 4.2 ± 2.7	per day
												Use EC: 2.41 ± 2.0	Use CC: 6.2 ± 4.5
												Dual use: 5.6 ± 2.5(CC), 2.3 ± 1.45 (EC)	Use EC: 15.6 ± 13.8
													Dual use: 8.0 ± 5.9 (CC), 14.7 ± 11.9 (EC)
Chaumont, M [16]	2019	Belgium	Population-base	Healthy smokers	25	Sham Vaping	N/A	5–10 min	Immediately	72	38	N/A	pack/years: 0.2 ± 0.5
Antoniewicz, L [2]	2019	Sweden	Population-base	Healthy smokers	15	EC without nicotine	19	30 min	Immediately/2 h/4 h/6 h	35.3	26	N/A	max 10 cigarettes/month
Tzortzi, A [17]	2018	Greece	Population-base	non-smokers	40	EC without activating	N/A	30 min	Immediately	50	24.6	N/A	N/A
Lappas, A. S [18]	2018	Athens	Population-base	Healthy smokers and asthmatic smokers	54	N/A	12	5 min	Immediately/15 min/30 min	59.3	23.7	N/A	pack/years: 2.0 ± 2.8
Kerr [19]	2018	UK	Population-base	Healthy smokers	20	CC	17.25	N/A	25 min	100	31.6	13 ± 11	per day: 7 ± 21.5
Staudt, M. R [20]	2018	USA	Population-base	non-smokers	10	EC without nicotine	N/A	30 min	2 h	50	40.2	N/A	N/A
Walele, T [21]	2018	UK	Population-base	Healthy smokers	209	N/A	N/A	2 years	1,3,6,12, 18,24 months	55	36.2	N/A	per day: 5–30
Palamidas, A [22]	2017	Greece	Population-base and hospital-base	Healthy smokers and non-smoker	76	EC without nicotine	11	N/A	25 min	41.6	58	N/A	per day: >15
Boulay, MÈ [23]	2017	Canada	Population-base	non-smokers	20	Placebo (no e-liquid)	N/A	1 hour	60 min	N/A	N/A	N/A	N/A
D’Ruiz, C. D [24]	2017	USA	Population-base	Healthy smokers	105	N/A	24	5 Days	5 Days	65	38	18.8 ± 10.8	per day: >15
Cravo, A. S [25]	2016	USA	Hospital-base	healthy smoker	408	CC	2.7	12 weeks	1, 2, 4, 6, 8, 10, 12 weeks	55.4	34.5	N/A	per day: 5–30
McRobbie, H [26]	2015	UK	Population-base	Healthy smokers	33	Dual use	9	4 weeks	Immediately	51.2	56.7	N/A	per day:
													Use EC: 16.3 ± 8.68
													Dual use: 21.0 ± 11.87
Pacifici, R [27]	2015	Italy	Population-base	Healthy smokers	34	Dual use	N/A	4 weeks	Immediately	52.9	42.6	22.0 ± 11.0	per day: 21.5 ± 9.0
Ferrari, M [28]	2015	Italy	Hospital-base	Healthy smokers	20	nicotine-free EC/CC	0.8	5 min	Immediately	70	36.2	N/A	pack/years: 19.4 ± 10.8
Marini, S [29]	2014	Italy	Population-base	Healthy smokers	25	CC/EC without nicotine	18	5 min	Immediately	56	28	N/A	N/A
Vardavas, C. I [30]	2012	Greece	Population-base	Healthy smokers	40	EC without the cartridge	11	5 min	Immediately	46.7	36	N/A	Minimum 5 pack/year

CC, conventional cigarettes; EC, electronic cigarette.

### Lung-Function Assessment

Our results showed no statistically significant change in pulmonary ventilation measures within 1 month of inhalation of e-cigarettes compared to pre-inhalation in participants ((healthy smokers, nonsmokers, or asthmatic smokers). (Table 2; Supplementary Table S4) [2, 16, 17, 20–27], but the decrease of FEV1/FVC% in the non-e-cigarette groups was more than that in the e-cigarette group (SMD: 1.18, 95%: 0.11–2.26) (Table 3; Supplementary Table S5) [2, 17, 20, 21]. Regarding the effects on pulmonary ventilation after 1 month and 3 months of e-cigarette inhalation, we assembled three indicators (FVC, FEV1 and PEF), none of which showed a statistically significant change (Table 4) [22, 24].

**TABLE 2:** Effects of electronic cigarette inhalation on lung function in different populations within 1 month (North America and Europe, 2012–2020).

Pulmonary function	Type of subjects	Number of studies	SMD (95%CI)	I^2^	*P* for heterogeneity
Pulmonary Ventilation Capacity					
FEV1 (L)					
	Healthy smokers	8	−0.73 (−2.52, 1.06)	0.99	0
	Asthmatic smokers	2	−0.0 (−0.62, 0.62)	—	—
	Non-smokers	1	−0.03 (−0.47, 0.41)	<0.01	0.92
	Overall	10	−0.60 (−2.10, 0.89)	0.99	0
FEV1 [%]					
	Healthy smokers	3	−0.10 (−0.45, 0.25)	<0.01	0.87
	Asthmatic smokers	1	−0.09 (−0.65, 0.46)	—	—
	Non-smokers	1	0.08 (−0.97, 1.12)	—	—
Overall		4	−0.08 (−0.37, 0.20)	<0.01	0.98
FVC(L)					
	Healthy smokers	6	−1.11 (−3.60, 1.37)	0.99	0
	Asthmatic smokers	2	−0.05 (−0.52, 0.42)	<0.01	0.84
	Non-smokers	1	0.00 (−0.62, 0.62)	—	—
	Overall	8	−0.76 (-2.53, 1.01)	0.99	0
FVC [%]					
	Healthy smokers	2	0.03 (−0.35, 0.41)	<0.01	0.91
	Asthmatic smokers	1	−0.05 (-0.61, 0.50)	—	—
	Non-smokers	1	0.00 (−1.05, 1.05)	—	—
	Overall	3	0.01 (-0.30, 0.31)	<0.01	0.99
FEV/FVC %					
	Healthy smokers	4	−0.10 (−0.42, 0.23)	<0.01	0.92
	Asthmatic smokers	2	−0.03 (−0.50, 0.44)	<0.01	0.7
	Non-smokers	2	0.32 (−0.22, 0.85)	<0.01	0.46
	Overall	6	−0.00 (−0.24, 0.24)	<0.01	0.9
PEF [l/s]					
	Healthy smokers	6	−0.91 (−2.74, 0.93)	0.99	0
	Asthmatic smokers	1	−0.10 (−0.65, 0.46)	—	—
	Overall	6	−0.79 (−2.41, 0.83)	0.99	0
PEF [%]					
	Healthy smokers	2	−0.23 (−0.61, 0.15)	<0.01	0.49
	Asthmatic smokers	1	−0.18 (−0.74, 0.37)	—	—
	Overall	2	−0.22 (−0.53, 0.10)	<0.01	0.78
TLC %					
	Healthy smokers	1	0.05 (−0.59, 0.61)	—	—
	Asthmatic smokers	1	−0.08 (−0.37, 0.37)	—	—
	Non-smokers	1	0.11 (−0.94, 1.16)	—	—
	Overall	2	0.00 (-0.37, 0.37)	<0.01	0.93
TV (L)					
	Healthy smokers	1	0.09 (−0.53, 0.71)	—	—
	Asthmatic smokers	1	0.29 (−0.59, 1.17)	—	—
	Overall	1	0.15 (−0.35, 0.66)	<0.01	0.71
O_2_ Saturation %					
	Healthy smokers	2	−0.40 (−1.30, 0.51)	0.83	0.02
	Asthmatic smokers	1	−0.32 (−1.16, 0.52)	—	—
	Non-smokers	2	−0.14 (−0.84, 0.55)	<0.01	0.72
	Overall	3	−0.31 (−0.71, 0.10)	0.37	0.18
Exhaled CO level					
	Healthy smokers	5			
	Overall	5	−1.48 (−2.82, −0.15)	0.93	0
FeNO					
	Healthy smokers	7	−1.07 (−2.17, 0.03)	0.95	0
	Asthmatic smokers	4	−0.09 (−0.42, 0.24)	<0.01	0.43
	Non-smokers	1	−0.34 (−1.14, 0.48)	—	—
	Overall	8	−0.66 (−1.32,-0.01)	0.92	0
Flow Resistance (IOS)					
Z5					
	Healthy smokers	2	0.29 (−0.10, 0.68)	<0.01	0.67
	Asthmatic smokers	2	0.48 (0.02, 0.93)	0.25	0.25
	Non-smokers	1	0 (−0.44, 0.44)	—	—
	Overall	3	0.28 (0.04, 0.51)	0.01	0.4
R5					
	Healthy smokers	3	0.16 (−0.18, 0.50)	<0.01	0.73
	Asthmatic smokers	3	0.42 (0.07, 0.78)	<0.01	0.61
	Non-smokers	2	−0.10 (−0.46, 0.26)	<0.01	0.44
	Overall	5	0.16 (−0.04, 0.37)	<0.01	0.5
R10					
	Healthy smokers	2	0.26 (−0.13, 0.64)	<0.01	0.8
	Asthmatic smokers	2	0.48 (0.08, 0.87)	0.32	0.23
	Non-smokers	1	0 (−0.31, 0.31)	—	—
	Overall	3	0.26 (0.03, 0.50)	1	0.4
R19					
	Healthy smokers	1	0.49 (−0.24, 1.22)	—	—
	Asthmatic smokers	1	0.13 (-0.75, 1.00)	—	—
	Non-smokers	1	−0.10 (−0.72, 0.52)	—	—
	Overall	2	0.14 (−0.27, 0.56)	<0.01	0.48
R5–19					
	Healthy smokers	1	−0.10 (−0.82, 0.61)	—	—
	Asthmatic smokers	1	0.78 (−0.14, 1.69)	—	—
	Non-smokers	1	−0.37 (−1, 0.25)	—	—
	Overall	2	0.04 (−0.46, 0.38)	0.52	0.12
R5–R20					
	Non-smokers	1	0.10 (−0.54, 0.34)	—	—
R20					
	Healthy smokers	2	0.18 (−0.20, 0.57)	<0.01	0.88
	Asthmatic smokers	2	0.46 (−0.08, 1.00)	—	—
	Non-smokers	1	0.06 (−0.25, 0.37)	<0.01	0.73
	Overall	3	0.16 (−0.06, 0.39)	<0.01	0.77
Fres					
	Healthy smokers	3	0.13 (−0.25, 0.52)	0.14	0.31
	Asthmatic smokers	2	0.22 (−0.29, 0.72)	0.13	0.29
	Non-smokers	1	−0.15 (−0.59, 0.26)	-	-
	Overall	4	0.08 (−0.16, 0.32)	0.02	0.4
X5					
	Healthy smokers	2	0.24 (−0.17, 0.65)	<0.01	0.6
	Asthmatic smokers	2	−0.08 (−0.65, 0.49)	0.26	0.24
	Non-smokers	1	−0.21 (−0.65, 0.23)	—	—
	Overall	3	−0.01 (−0.26, 0.24)	0.02	0.4
X20					
	Healthy smokers	1	1.98 (1.32, 2.64)	—	—
	Asthmatic smokers	1	−0.60 (−1.15, −0.05)	—	—
	Overall	1	0.68 (−1.84, 3.21)	0.97	0
AX					
	Healthy smokers	3	0.15 (−0.25, 0.56)	0.2	0.29
	Asthmatic smokers	2	0.19 (−0.43, 0.82)	0.38	0.2
	Non-smokers	1	−0.07 (−0.51, 0.36)	—	—
Overall		4	0.12 (−0.13, 0.36)	0.04	0.39

FEV1, forced expiratory volume in 1 s; FVC, forced vital capacity; FEV1/FVC%, forced expiratory volume in one second to forced vital capacity; PEF, peak expiratory flow; TV, tidal volume; TLC, total lung capacity; Exhaled CO level, exhaled carbon monoxide level; FeNO, fractional exhaled nitric oxide; Z5Hz, respiratory impedance at 5 Hz; R5Hz, R10Hz, R20Hz, respiratory resistance at 5, 10 and 20 Hz; R5–19 Hz, the difference of R5 Hz and R19 Hz; R5–20 Hz, the difference of R5 Hz and R20 Hz; fres, resonant frequency; X5, respiratory system reactance at 5 Hz; X20, respiratory system reactance at 20 Hz; AX, reactance area.

**TABLE 3:** Effects of electronic cigarette on pulmonary function compared with control groups (North America and Europe, 2012–2020).

Pulmonary function	Types of controls	Number of studies	SMD (95%CI)	I^2^	P for heterogeneity
Pulmonary Ventilation Capacity
FEV1 (L)	Non-users	4	0.05 (−0.27, 0.36)	—	—
	Cigarette	2	0.02 (−0.29, 0.33)	<0.01	0.98
	Overall	5	0.03 (−0.19, 0.25)	<0.01	0.87
FEV1 [%]	Non-users	2	0.28 (−0.75, 1.30)	0.51	0.15
	Cigarette	2	0.02 (−0.17, 0.21)	<0.01	0.85
	Overall	3	0.03 (−0.15, 0.21)	<0.01	0.67
FVC(L)	Non-users	3	0.23 (−0.40, 0.86)	66.1	0.052
	Cigarette	4	−0.04 (−0.22, 0.14)	<0.01	0.972
	Overall	5	0 (−0.17, 0.17)	0.03	0.41
FVC [%]	Non-users	2	−0.07 (−0.56, 0.42)	<0.01	0.39
	Cigarette	1	−0.07 (−0.44, 0.30)	<0.01	0.76
	Overall	2	−0.07 (−0.36, 0.23)	<0.01	0.84
FEV/FVC %	Non-users	3	2.73 (−1.01, 6.47)	0.97	<0.01
	Cigarette	2	0.06 (−0.28, 0.43)	<0.01	0.98
	Overall	4	1.18 (0.11, 2.26)	0.92	<0.01
PEF [l/s]	Non-users	1	−0.03 (−0.56, 0.49)	—	—
	Cigarette	3	−0.08 (−0.22, 0.07)	<0.01	0.9
	Overall	3	−0.08 (−0.21, 0.06)	<0.01	0.96
PEF [%]	Non-users	1	−0.18 (−0.7, 0.35)	—	—
	Cigarette	1	0.15 (−0.22, 0.52)	<0.01	0.78
	Overall	1	0.04 (−0.26, 0.34)	<0.01	0.59
MEF25 [l/s]	Non-users	1	0.02 (−0.5, 0.55)	—	—
	Cigarette	1	−0.11 (−0.49, 0.26)	<0.01	0.461
	Overall	1	−0.07 (−0.37, 0.24)	<0.01	0.7
TV (L)	Non-users	1	−0.08 (−0.7, 0.54)	—	—
O2 Saturation %	Non-users	3	0.20 (−0.22, 0.63)	<0.01	0.4
	Cigarette	1	0.40 (0.04, 0.76)	<0.01	0.4
	Overall	3	0.32 (0.04, 0.59)	<0.01	0.55
Exhaled CO level	Non-users	2	0.13 (−0.29, 0.55)	<0.01	0.8
	Cigarette	5	−0.84 (−1.73, 0.05)	0.89	<0.01
	Overall	5	−0.54 (−1.18, 0.11)	0.86	<0.01
FeNO	Non-users	7	−0.13 (−0.40, 0.13)	<0.01	0.58
	Cigarette	3	0.24 (−0.21, 0.68)	0.59	0.06
	Overall	7	0.03 (−0.22, 0.27)	0.34	0.14
Flow Resistance (IOS)					
Z5	Non-users	2	0.35 (−0.55, 1.25)	0.77	0.04
R5	Non-users	4	0.17 (−0.29, 0.63)	0.55	0.08
R10	Non-users	2	0.31 (−0.41, 1.02)	0.55	0.09
R19	Non-users	2	0.16 (−0.56, 0.88)	0.56	0.13
R5–19	Non-users	2	0.10 (−1.37, 0.57)	<0.01	0.83
R5–R20	Non-users	1	0.10 (-0.54, 0.34)	—	—
R20	Non-users	2	0.29 (−0.25, 0.84	0.43	0.19
Fres	Non-users	3	−0.11 (−0.43, 0.21)	<0.01	0.77
X5	Non-users	3	−0.15 (−0.47, 0.17)	<0.01	0.49
X10	Non-users	1	—	—	—
X20	Non-users	1	—	—	—
AX	Non-users	3	−0.13 (−0.45, 0.19)	<0.01	0.89

FEV1, forced expiratory volume in 1 s; FVC, forced vital capacity; FEV1%/FVC, forced expiratory volume in one second to forced vital capacity; PEF, peak expiratory flow; MEF25, maximal expiratory flow at 25% of FVC; TV, tidal volume; TLC, total lung capacity; Exhaled CO level, exhaled carbon monooxide level; FeNO, fractional exhaled nitric oxide; Z5Hz, respiratory impedance at 5 Hz; R5Hz, R10Hz, R20Hz, respiratory resistance at 5, 10 and 20 Hz; R5–19 Hz, the difference of R5 Hz and R19 Hz; R5–20 Hz, the difference of R5 Hz and R20 Hz; fres, resonant frequency; X5, respiratory system reactance at 5 Hz; X20, respiratory system reactance at 20 Hz; AX, reactance area.

**TABLE 4:** Effects on lung function after 1 month and 3 months of electronic cigarette inhalation (North America and Europe, 2015–2016).

Pulmonary function	Author	Baseline		3 Month				SMD(95%)	I^2^	*P* for heterogeneity
		Number	Mean	sd	*n*	Mean	sd			
FVC										
	Cravo, A. S	306	4.73	1.01	286	−0.12	0.27	−6.46 (−6.86, 6.06)		
	Walele, T	110	4.68	1.01	96	4.45	1.27	−0.16 (−0.43, 0.11)	99.8	<0.01
								−3.31 (−9.48, 2.87)		
FEV1										
	Cravo, A. S	306	3.64	0.79	286	-0.09	0.04	−6.56 (−6.96, −6.15)		
	Walele, T	110	3.60	0.79	96	3.54	0.75	−0..08 (−0.35, 0.20)	99.9	<0.01
								−3.23 (−9.67, 3.04)		
PEF										
	Cravo, A. S	306	495.57	125.89	286	11.7	75.58	−4.62 (−4.93, 4.31)		
	Walele, T	110	513.02	114.62	96	511.69	114.19	−0.01 (−0.29, 0.26)	99.8	<0.01
								−2.32 (−6.84, 2.20)		

FVC, forced vital capacity; FEV1, forced expiratory volume in 1 s; PEF, peak expiratory flow.

After inhalation of e-cigarettes, the decrease in O_2_ saturation was not statistically significant in the e-cigarette group (Table 2; Supplementary Table S4) [17, 26, 28], but the degree of decline was less than in the cigarette groups (SMD: 0.40, 95%: 0.04–0.76), especially when compared with the conventional cigarette group (SMD: 0.32, 95%: 0.04–1.08) (Supplementary Table S5) [17, 26, 28]. Exhaled CO (SMD: −1.48, 95%: −2.82–0.15) was significantly lower in healthy smokers after e-cigarette inhalation. Although there was a statistically significant reduction in FENO (SMD (−0.66, 95%: −1.32, −0.01) in the overall participants after vaping, the clinical significance was weak. No statistically significant reductions were observed in the subgroups (healthy smokers, nonsmokers, and asthmatic smokers), either because there was indeed no effect of e-cigarette inhalation on FENO within 1 month in each subgroup or because of the small sample size. (Table 2; Supplementary Table S4) [2, 16, 17, 19–21, 25, 28–31]. Nonetheless, the degree of the decline evinced no significant difference when compared to the control group [2, 17, 20, 21, 25, 28–30, 32, 33] (Supplementary Table S5).

**FIGURE 2:**
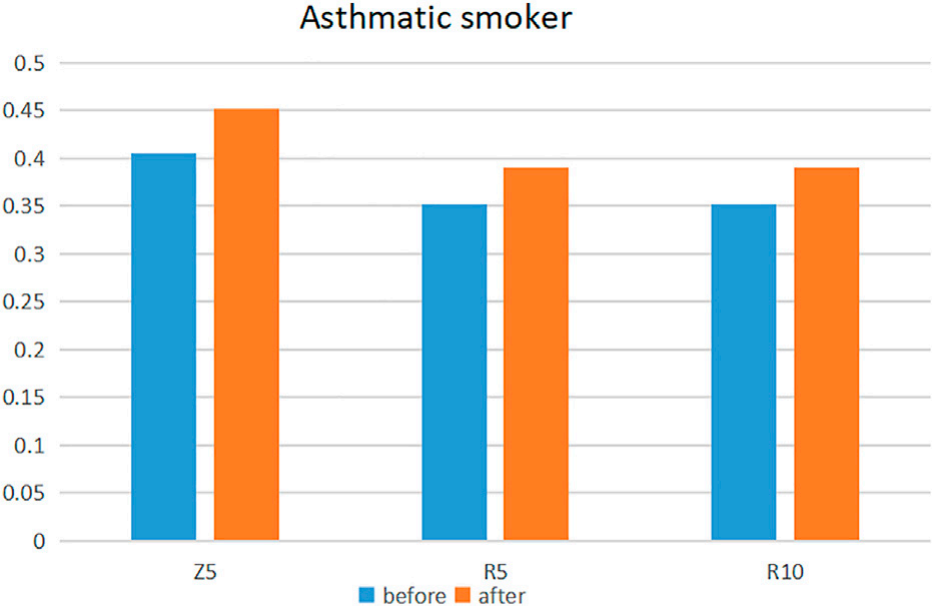
Changes in flow resistance in asthmatic smokers before and after e-cigarette use (Europe, 2018–2020).

A statistically significant increase in flow resistance, after e-cigarette inhalation, was apparent only among asthmatic smokers (Z5: SMD: 0.48, 95%: 0.02–0.93; R5: SMD: 0.42, 95%: 0.07–0.78 and R10: SMD: 0.48, 95%: 0.08–0.87) (Figure 2; Table 2; Supplementary Table S4) [2, 16, 19, 20, 31]. Compared to the non-e-cigarette groups, the degree of increase was not significantly different [2, 20, 31, 32, 34] (Table 3; Supplementary Table S5).

### Sensitivity Analyses and Publication Bias

Sensitivity analyses and publication-bias evaluations were performed for statistically significant indicators, including FEV1/FVC, O_2_ saturation, CO, FeNO, Z5 and R10. The sensitivity analysis showed that the results of these indicators were stable (Supplementary Table S6).

Begg’s and Egger’s tests for O_2_ saturation, CO, FeNO, Z5 and R10 showed no publication bias. Conversely, the results for FEV1/FVC showed some bias. Nevertheless, after adjustment by the trim-and-fill method, the results showed that the decrease of FEV1/FVC was still significantly smaller in the e-cigarette group. The FEV1/FVC result was not affected by the publication bias (SMD: 3.27 95%: 1.11–9.62) (Supplementary Table S7).

## Discussion

Cigarette smoking is an important cause of lung cancer, acute fatal complications of atherosclerotic cardiovascular disease, and chronic obstructive pulmonary disease (COPD) [34]. The World Health Organization (WHO) Framework Convention on Tobacco Control (FCTC) advises that the key to reducing the health burdens associated with tobacco is to encourage abstinence among smokers [35]. Indeed, surveys indicate that most smokers would like to quit. Unfortunately, smoking is a very difficult addiction to break, even for those with a strong desire to do so. Until recently, smokers were presented with two stark choices, namely, quitting smoking or suffering from the harmful effects of continued smoking. Now, however, smokers have a third choice: tobacco-harm reduction [36].

E-cigarettes, as a substitute for conventional cigarettes, are advertised as reducing the harmful effects of tobacco. In the original registered patent, it was claimed that the main advantage of the e-cigarette device is that it enables “smoking” without tar (tar being the main source of harmful substances in tobacco), which in turn significantly reduces cancer risk [3]. Meanwhile, the e-cigarette liquid may contain various flavourings especially attractive to children or adolescents [37]. According to the FDA, the marketing of e-cigarettes has been directed at young adults and children, and the use of these products in this population is rapidly increasing [38].

In 2019, moreover, the United States reported an outbreak of EC-related disease, which the Centers for Disease Control (CDC) have designated “E-cig and Vaping Acute Lung Injury” (EVALI) [39]. This has focused attention on the issue of whether e-cigarettes are safe to use, especially since, despite their short time on the market, these devices have flourished as a supposedly safe way to quit smoking [1]. The fact is, thus far, there is limited understanding of the effects of e-cigarette inhalation on lung health. This study therefore considered the effects of e-cigarettes on lung function from two perspectives.

### What Were the Differences Between the E-Cigarette Users and the Cigarette Smokers and Non-Users Groups?

Our results showed that there was no significant difference in lung ventilation between the e-cigarette use group and the cigarette users or non-use groups. In terms of the existing evidence, e-cigarette inhalation may not alter lung ventilation. Even if it does so, the effect is no more harmful than that of conventional cigarette users groups. Compared with the e-cigarette group, the degree of O_2_ saturation decrease differed more significantly in the cigarette group, and particularly in the traditional cigarette group. This effect may be caused by carbon-monoxide emissions during smoking [16]. In summary, short-term e-cigarette inhalation did not significantly affect lung function compared to smoking, and the long-term effects need to be further studied.

### What are the Changes in Lung Function After Exposure to E-Cigarettes in Different Populations?

Based on the relevant pulmonary ventilation indicators provided by the studies we addressed, it appears that short-term e-cigarette inhalation had no significant impact on lung ventilation in different population, which is consistent with the conclusions of previous studies [20, 21]. Nonetheless, this may be because the history of e-cigarette inhalation is simply too brief and recent, so far, to afford insights into lung-function effects. Indeed, the fact that no effect on pulmonary ventilation function was observed after 3 months of e-cigarette inhalation may reflect a lack of relevant studies. We merely collected relevant indicators from two studies [22, 24], and this does not sufficiently illuminate putative changes of pulmonary ventilation function after the long-term use of e-cigarettes.

We observed a significant reduction in FeNO after e-cigarette inhalation. Although clinically insignificant, the statistically significant decline is consistent with previous findings. Nitric oxide is a widely studied marker of respiratory diseases [40], and a lower level of FeNO is associated with decreased respiratory function [41]. Previous studies have suggested that this may be due to the oxidative stress caused by inhaling e-cigarettes, while the introduction of toxic or irritating substances (degraded by e-cigarettes) into the lungs interferes with pulmonary homeostasis [7]. Further research is needed to investigate the relationship between inhaling e-cigarettes and exhaled nitric oxide.

As with FeNO, we also observed a significant decrease in exhaled CO after e-cigarette use in healthy smokers. CO is a toxic gas known to be generated in high concentrations during cigarette combustion, and exhaled carbon monoxide has been widely used as a biomarker of exposure to cigarette smoke. E-cigarette use is unaffected by the combustion process, so it is not surprising that a significant correlation has been observed with reduced exhaled CO levels from baseline, following e-cigarette usage [4]. In summary, however, e-cigarette inhalation may have some physiological effects on the ability of the lungs to diffuse, even for short periods of time.

We noted that pulse oscillations detected a significant airway obstruction response (a statistically significant increase in Z5 and R10) in asthmatic patients who inhaled e-cigarettes, while spirometry did not demonstrate any change. In both healthy individuals and asthmatics, increases in Z5 and R10 are associated with acute bronchoconstriction and reduced airway diameter. Although asthma comprises inherent bronchial hyperreactivity, peripheral airway obstruction after e-cigarette inhalation may represent a superimposed effect of the e-cigarette and hyperreactivity, leading to more intense bronchoconstriction, in the same manner as inhalational asthma [19]. The patients in our study took their medication regularly according to the GINA guidelines and were well controlled. Medications may also influence our results, although no studies have investigated the effects of e-cigarettes on patients using related asthma medications (inhaled corticosteroids, etc.). Asthma is a disease with its own variability (pollen season, infection and mold exposure) [42], but airway obstruction due to vaping cannot be ignored in combination with our results.

Airway obstruction may be caused by the electronic-cigarette liquid, or more specifically, by propylene-glycol irritation and inflammation of the airway and lungs; in fact, mild airway obstruction can occur even in non-asthmatic individuals [19]. Nevertheless, there is no extant evidence regarding a causal relationship between e-cigarettes and asthma, and more research is needed to verify whether e-cigarettes are suitable for patients with asthma. Based on current evidence, some clinicians and researchers still advocate that smokers with asthma should switch to e-cigarettes to mitigate the role of smoking in asthma exacerbation [43]. In reality, one should note that there are many “sensitive” cohorts in the e-cigarette market and audience, such as teenagers and asthmatics. In particular, the level and proportion of e-cigarette use among adolescents is increasing [44]. At the same time, adolescents also evince a high incidence of asthma. Therefore, one should strengthen the relevant health-related publicity, *and* the education of adolescents (especially those who have symptoms of wheezing), giving such individuals appropriate health guidance as required [43].

Overall, effects on pulmonary ventilation, pulmonary diffusing capacity and flow resistance are *not* worse after individuals switch to e-cigarettes, but further studies are needed to determine whether e-cigarette usage is effective in quitting smoking. Even if there are negative effects, the latter will not be unduly serious compared to traditional cigarettes. There may even be improvements in lung function after switching from cigarettes to e-cigarettes. Our results are consistent with those of one long-term study, which showed that lung function did not deteriorate after switching to e-cigarettes [22].

As noted earlier, the current e-cigarette audience is relatively young and broad. In addition to focusing on adolescents, nonetheless, we should pay close attention to the effects of e-cigarettes on high-risk populations, i.e., those who are especially vulnerable to the effects of cigarette smoking, including asthmatic patients and those suffering from chronic obstructive pulmonary disease, as well as pregnant women [4]. With e-cigarette usage becoming increasingly prevalent, we need further to investigate the impact of e-cigarettes on lung function in vulnerable populations.

The strengths of our study include the fact that it is the first comprehensive analysis of the effects of e-cigarette inhalation in terms of lung ventilation, lung-diffusion capacity and flow resistance. Second, we collected and distinguished the effects of short-term, 1 month, and 3 month e-cigarette inhalation on lung function. Third, we assembled various types of control group to explore the differences between the e-cigarette groups and non-e-cigarette groups.

Conversely, the study does evince some limitations. First, research on the effect of long-term e-cigarette inhalation on lung function remains incomplete, because the relevant research, so far completed, is insufficient to support a comprehensive analysis. Second, the stability of the results may be affected by the different smoking patterns, e-cigarette types, exposure times and nicotine-content levels included in the studies. Third, we included a total of 18 studies, most of which had comparatively few participants, so we could obtain only relatively limited information. Further studies, with larger samples, will be needed in future.

Our current results indicate that *short-term* e-cigarette inhalation may not have a significant effect on lung function. The effects of *long-term* e-cigarette inhalation on lung function, by contrast, merit long-term clinical observation, and we require additional longitudinal studies for assessment. In addition, more clinical trials are needed to evaluate the efficacy of e-cigarette usage as a smoking-cessation tool.
